# Intra-articular administration of extra-virgin olive oil in degenerative osteoarthritis

**DOI:** 10.1186/s13018-024-04818-5

**Published:** 2024-06-08

**Authors:** Ahmet Pamiry, Mehmet Yiğit Gökmen, Mustafa Tekin

**Affiliations:** 1 Department of Orthopedics and Traumatology, University of Health Sciences, Adana City Training and Research Hospital, Adana, Türkiye; 2 Department of Orthopedics and Traumatology, Çukurova University Faculty of Medicine, Adana, Türkiye

**Keywords:** Osteoarthritis, Polyphenols, Intra-articular injection, Olive oil

## Abstract

**Background:**

We aimed to analyze the outcomes of intraarticular extra virgin olive oil (EVOO) injection on mechanically induced rabbit knee osteoarthritis (OA) by studying the morphological, histological, and radiological findings.

**Methods:**

The study was conducted on 32 New Zealand White rabbits. The randomly numbered subjects were divided into two main groups. The rabbits numbered 1 to 16 were selected to be the group to receive EVOO, and the remaining were selected into a control group. Both groups were separated into two subgroups for short-term (five weeks) and long-term (10 weeks) follow-up. Anterior cruciate ligament transection was applied on the left knees of all the rabbits via medial parapatellar arthrotomy to simulate knee instability. Immediately after the surgical procedure, 0.2 cc of EVOO was injected into the knee joint of rabbits numbered 1–16, and the control group received 0.2 cc of sterile saline. On the 14th day, long-term group subjects were administered another dose of 0.2 cc EVOO intraarticularly.

**Results:**

The gross morphological scores of the control group subjects were significantly different from the EVOO group for both short-term (*p* = 0,055) and long-term (*p* = 0,041) scores. In parallel, the MRI results of the EVOO subjects were significantly different from the control group for both short-term and long-term follow-up assessment scores (*p* = 0.017, *p* = 0.014, respectively). The Mankin scoring results showed that there were statistically significant differences between the EVOO and control group in the comparison of both total scores (*p* = 0.001 for short-term and *p* = 0.004 for long-term) and subgroup scoring, including macroscopic appearance, chondrocyte cell number, staining, and Tidemark integrity in both short-term (*p* = 0.005, *p* = 0.028, *p* = 0.001, *p* = 0.005, respectively) and long-term assessments (*p* = 0.002, *p* = 0.014, *p* < 0.001, *p* = 0. 200, respectively).

**Conclusions:**

We have observed promising outcomes of intra-articular application of extra virgin olive oil in the treatment of acute degenerative osteoarthritis in rabbit knees. Due to its potential cartilage restorative and regenerative effects, EVOO, when administered intra-articularly, may be a promising agent to consider for further research in the treatment of OA.

## Introduction

Osteoarthritis (OA) is one of the most common progressive diseases affecting synovial joints and occurs highly prevalent in people over 60 years of age [1]. With no definitive cure to date, the mechanism of progression of this debilitating disease remains largely mysterious and, therefore, requires a more personalized approach to its management. Current treatments aim to reduce the symptoms of inflammation due to the damage in the underlying articular tissue. However, such approaches do not address the problems, including pain directly caused by OA or the mobility and activity limitations often reported. The popular nutritional routines, especially the traditional Mediterranean diet, consisting of vegetables, fruits, nuts, fish/fish oil, and cereals, help avoid common chronic conditions correlated with many inflammatory conditions [2]. The Osteoarthritis Initiative study indicates that staying on the Mediterranean style nutrition was linked to improved life quality with a significantly lower prevalence of OA [3]. Considering the parallel increase in life expectancy and the risk of developing OA, an approach based on dietary modification appears to be advantageous in terms of risk/benefit and is potentially more feasible [4]. The Mediterranean diet is rich in extra-virgin olive oil (EVOO) as the primary lipid source resulting in a high intake of phytonutrients, containing natural phenols, especially. Plant-sourced phenols are inessential plant metabolites and exhibit antioxidant, anticarcinogenic, and anti-inflammatory properties [5]. The number of studies which are suggesting that the nutraceutical EVOO compounds that are polyphenol-rich induce antiinflammatory processes and lessen cartilage degradation is increasing [6]. Moreover, reports highlight that the polyphenols extracted from EVOO demonstrate significant anti-inflammatory properties and play important roles in the maintenance of the articular cartilage [7].

The treatment options vary, including oral medications, intraarticular applications, and surgery. The reports show that intra-articular applications, when compared to oral treatment alternatives, are more effective, supporting the fact that currently, intraarticular treatment of OA is the best option in mild to moderate cases including hyaluronic acid (HA), platelet-rich plasma (PRP), and stem cell applications [8]. Not long ago, researchers began using EVOO components intraarticularly and reported successful results [9–11].

In parallel to all researchers, aiming to provide an accessible, available, and cheap alternative intraarticular agent for OA, we aimed to analyze the outcomes of intraarticular EVOO injection on mechanically induced rabbit knee OA by studying the morphological, histological, and radiological findings.

## Methods

The animal experiment study was conducted at Çukurova University Health Sciences Experimental Application and Research Center (SABIDAM) between 20.09.2021 and 30.11.2021. This study was approved by the local ethics committee of Çukurova University Health Sciences Experimental Application and Research Center (SABİDAM) on 15.01.2021 and was conducted according to the current regulations for the care of laboratory animals at the Çukurova University. The experiments were blinded to all researchers involved in the animal experiments.

### The test subjects

Because of their similarity to human anatomy and physiology, small size, easy applicability of surgical procedures, and low cost, 32 New Zealand White rabbits were selected as test subjects. The average age of the subjects was six months, reneged between 5 and 6.5 months, and their average weight was 3000 g (minimum 3 kg and maximum 3.5 kg). The rabbits were kept under stable conditions of temperature (22–25 °C), humidity (40–70%), exposed to light for 12 h per 24 h, and food and water accessible ad libitum. All subjects were quarantined in a shelter cage at the SABIDAM for three weeks to rule out Tularemia disease.

### The reagent

Extra virgin olive oil (EVOO) was obtained from local producers to ensure the authenticity, polyphenol saturation, and freshness of the reagent. The EVOO was filtered through micron filters sized 0.6 and 0.45, respectively, to provide a reagent purified from pathogens. Then, samples taken from the EVOO were incubated for fungal and bacterial microbiological studies, and no growth of any pathogens was observed on the fifth day of incubation.

### Grouping and preliminary examination

The subjects were randomly numbered from 1 to 32 and divided into two main groups. The rabbits numbered 1 to 16 were selected to be the group to receive EVOO, and the remaining were selected into a control group. Both groups were separated into two subgroups for short-term (five weeks) and long-term (10 weeks) follow-up. The subgrouping was also selected by randomization.

After the end of the quarantine monitoring on the 22nd day, all rabbits were weighed on an electronic scale, and the anesthesia dose was adjusted precisely. Ketamine hydrochloride 10% (50 mg/kg, intraperitoneal Ketasol®, Richter Pharma AG, Wels Austria) and Xylazine hydrochloride (10 mg/kg, intraperitoneal) were administered combined in the same injection via the groin area of each rabbit for intraperitoneal sedation. Following sedation, magnetic resonance imaging (3T PHILIPS IMEGMIA) was performed on the right knees of all rabbits, in T1 and T2 sequences in coronal and sagittal planes, in 14–16 slices. The images were sent to the radiology department for blind radiological evaluation.

### The surgery

**Fig. 1:**
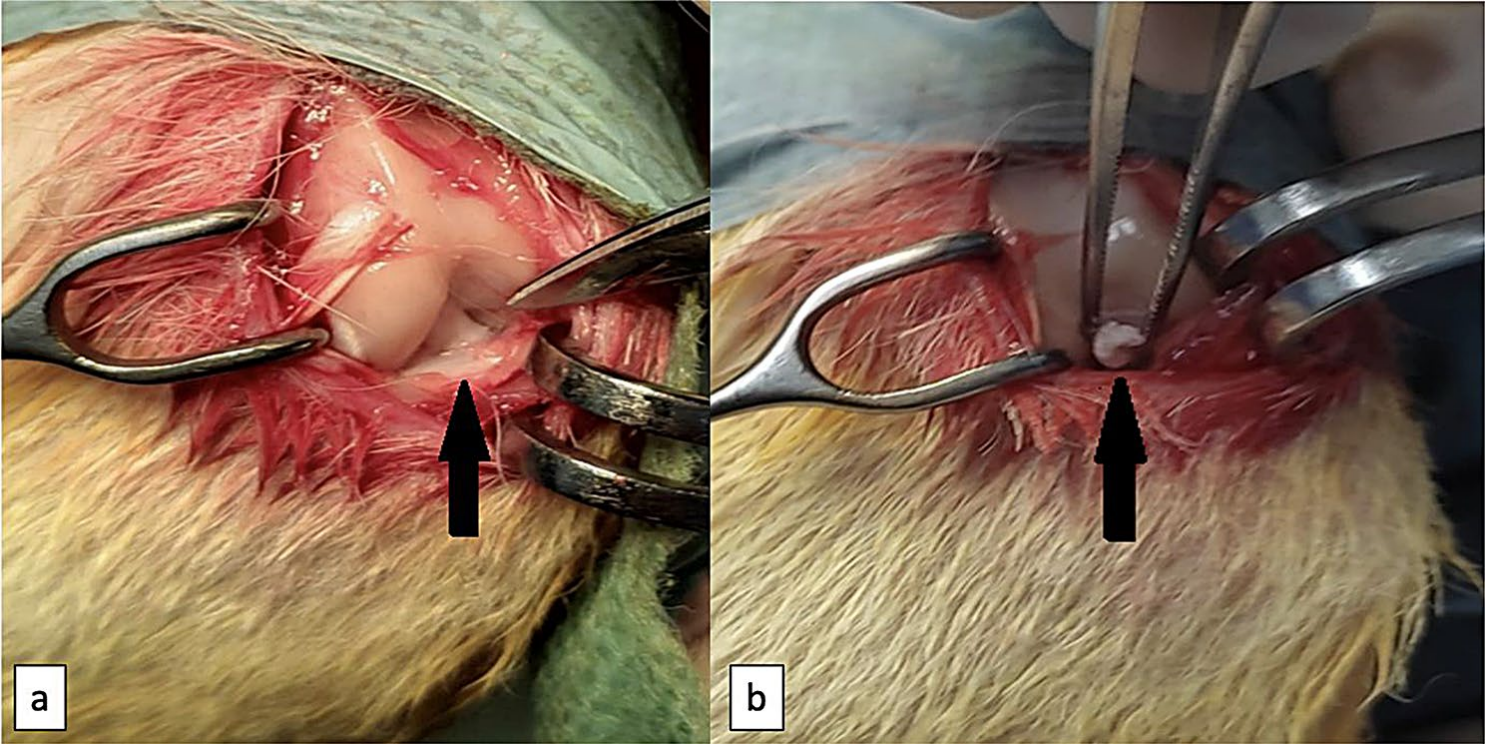
Anterior cruciate ligament transection was applied on the left knees of all the rabbits via medial parapatellar arthrotomy to simulate knee instability (**a**)The intact anterior cruciate ligament (**b**)After the anterior cruciate ligament transection

All rabbits were weighed with an electronic scale before surgery, and the anesthesia dose was calculated separately for each subject. Ketamine hydrochloride 10% (50 mg/kg, intraperitoneal Ketasol®, Richter Pharma AG, Wels Austria) and Xylazine hydrochloride (10 mg/kg, intraperitoneal Xylasin Bio 2%, Bioveta, İntermed Ecza Deposu Import, Export, Trade and Contracting Ltd. Ankara) were administered together in the same injection, via the groin area of each rabbit, and anesthesia was achieved. Following the shaving of the surgical area, the subjects were placed in the supine position on the operating room table and covered sterilely after cleaning the surgical field with Povidone-iodine (Batticon®, Adeka İlaç Sanayi ve Ticaret A.Ş. Samsun) solution. Using the method described by Yoshioka et al. [12], rabbits were entered from the left knee through a medial parapatellar mini-incision, and the layers were passed. The patella was slightly lateralized and the joint was placed in full flexion. The ACL was fully visible and cut with the help of a number 11 scalpel (Fig. 1). Later, forward translation of the tibia was observed during the anterior drawer test. Subdermal tissue was repaired primarily with 4 − 0 Vicryl (Coated VICRYL®, Ethicone) and dermis with 4 − 0 Ethilon (Prolene®, Ethicone, Johnson&Johnson) suture. Immediately after the surgical procedure, 0.2 cc of EVOO was injected into the knee joint of rabbits numbered 1–16 through the incision line. The control group received 0.2 cc of sterile saline. Following injection, all subjects were wrapped in a sterile dressing. On the 14th day, long-term group subjects were administered another dose of 0.2 cc EVOO intraarticularly to the same knee using the same method.

### Follow-up

No immobilization method was applied after surgery, and the subjects were allowed to roam freely in the shelter cages and did not have mouth masks. At the first postoperative day visit, it was observed that they were biting off their dressings. The subjects in the polyphenol group were mobilized on all four legs with a slight limp from the first postoperative day. On the contrary, similar mobilization characteristics in the control group were not observed until the third postoperative day. On the 14th day of the surgery, the polyphenol group subjects were administered a second dose of 0.2 cc of EVOO to the knee joint.

During the 10-week follow-up period, no wound site complications (e.g. infection) were observed in either group, but two of the test subjects, one from each group, died. The first loss was subject number eight of the polyphenol group, which died during the surgery, and the second loss was subject number 32 from the control group. The autopsy revealed that the loss of number 32 was due to coccidiosis. The data of the deceased subjects was excluded from the analysis. The control group was kept in separate cages during the study. All subjects of the control group only were administered coccidiosis prophylaxis based on veterinary approval since there was no physical contact during the remaining course of the study.

On the 36th postoperative day, short-term subgroup subjects from each group were selected for MR imaging of the knee joints. Ketamine hydrochloride 10% (50 mg/kg, intraperitoneal Ketasol®, Richter Pharma AG, Wels Austria) and Xylazine hydrochloride (10 mg/kg, intraperitoneal) were administered combined in the same injection via the groin area of each rabbit for intraperitoneal sedation. Following sedation, magnetic resonance imaging (3T PHILIPS IMEGMIA) was performed on the right knees of all rabbits, in T1 and T2 sequences in coronal and sagittal planes, in 14–16 slices. The same imaging procedure was applied for long-term subgroup rabbits of both groups on the 71st postoperative day.

### Morphological, histological, and radiological assessment

The short-term group members were sacrificed on the 36th day of the procedure, and long-term group members were sacrificed on the 71st day of the procedure, the day following the control MRI, using intracardiac potassium administration. No complication findings and suture materials were observed at the incision site of any test subjects. A new incision was made through the old incision scar. Following arthrotomy, the patellar tendon was cut from the tibial attachment site using a number 15 scalpel, and by lifting the joint proximally, maximum exposure was achieved. Then, the joint was flexed, and the findings of induced OA following ACL incision, including the gross morphologic changes of both condyles of the femur and patellar fossa, were recorded. The assessment of the gross morphologic changes due to induced OA was performed according to a modified macroscopic articular cartilage damage grading criteria initially described by Collins et al., which indicates the severity and macroscopic morphologic changes of cartilage damage due to degenerative osteoarthritis on the articular surface of the human talus. The grading system was classified into five grades: Grade 0, indicating no evidence of degenerative morphologic change; Grade 1, describing early fibrillation, flaking, shallow pits or grooves, and small bubbles developed on the cartilage surface with no alterations in articular surface geometry; Grade 2 representing deep fibrillation and fissuring, flaking, pitting and/or bubbling, early marginal hyperplasia and small osteophytes; Grade 3 signifying extensive fibrillation and fissuring, obvious osteophytes, including 30% or less of the articular cartilage surface eroded to the subchondral bone; and Grade 4 indicating prominent osteophytes, lips or shelves at the articular margin, greater than 30% of the articular surface eroded to the subchondral bone, and gross geometric changes [13].

The histological assessment began with removing the remains of soft tissue from the femur using a scalpel, preserving the distal articular cartilage. The proximal femur was osteotomized from the supracondylar region using a saw and was placed in 10% formaldehyde solution for histological evaluation.

The MRI images of the knee joint of all rabbits were evaluated by the same blinded expert radiologist. Radiologically, articular cartilage was evaluated using the osteochondral lesion MRI staging criteria of Dipaola et al. [14] The grading system was divided into four grades: Grade I, showing articular cartilage intact but thickened with small intensity changes; Grade II, indicating a break in the articular cartilage with a rim of low signal intensity on T2-weighted sequences behind the fragment, suggesting fibrous attachment or granulocytic tissue, Grade III, with a break in the articular cartilage with high-intensity changes behind the fragment on T2-weighted sequences, suggesting fluid behind the lesion, and Grade IV, expressing loose bodies with a defect of the articular cartilage surface.

Regarding the histological assessment, the specimens placed in boxes containing 10% neutral formol solution were delivered to the laboratory of the Department of Histology, Çukurova University Faculty of Medicine. The macroscopic assessment and the histopathological scoring of all the specimens were performed by the same blinded specialized histologist. The histologic and histochemical changes in the cartilage tissue were scored according to the histopathologic scoring system described by Mankin et al. by considering cartilage structure, cellularity, staining, and tidemark integrity. Each item is scored separately, and the total score is ranged between 0 and 14. The maximum score for normal articular cartilage is 1, and the score ranges for mild, moderate, and severe osteoarthritic changes are two and five, six and nine, and 10 and 14, respectively. Although the histological assessment in the Mankin scoring system is based on Safranin O staining, the use of Alcian blue is widely accepted [15, 16]. We used Alcian blue in our study.

### Statistics

The statistical analysis was performed using the SPSS software (IBM Corp. Released 2011. IBM SPSS Statistics for Windows, Version 20.0. Armonk, NY: IBM Corp). The categorical measurements, such as macroscopic appearance and tidemark integrity, were summarized as numbers and percentages, and the total histology score was summarized as median and IQR. Chi-square test statistics were used to compare categorical measurements between groups. The normal distribution of the histology total scores was tested using the Shapiro-Wilk test. Mann Whitney U test was used to compare the histology total scores, but it did not show a normal distribution between the EVOO and control groups. IBM SPSS Statistics Version 20.0 package program was used for statistical data analysis. The statistical significance level was set as 0.05 in all tests.

## Results

The gross morphological scoring results showed that the scores of the control group subjects were significantly different from the EVOO group for both short-term (*p* = 0,055) and long-term (*p* = 0,041) scores. The lack of grade 1 subjects in both of the subgroups of the control group was noteworthy (Table 1).

**Table 1:** The summary of the morphological, histological, and radiological results

		Short-term follow-up			Long-term follow-up		
		EVOO *n* (%)	Control *n* (%)	*p*	EVOO *n* (%)	Control *n* (%)	*p*
MRI grading	Grade 1 Grade 2 Grade 3 Grade 4	6 (86) 1 (14) 0 (0) 0 (0)	1 (14) 0 (0) 3 (43) 3 (43)	**0.017**	4 (57) 3 (43) 0 (0) 0 (0)	0 (0) 4 (57) 0 (0) 3 (43)	**0.041**
Gross morphology grading	Grade 1 Grade 2 Grade 3 Grade 4	4 (57) 3 (43) 0 (0) 0 (0)	0 (0) 3 (43) 1 (14) 3 (43)	**0.055**	1 (14) 4 (57) 2 (29) 0 (0)	0 (0) 3 (43) 1 (14) 3 (43)	**0.041**
Mankin (Histological) overall scoring	Normal Mild Moderate Severe	4 (57) 3 (43) 0 (0) 0 (0)	0 (0) 0 (0) 3 (43) 4 (57)	**0.001**	2 (29) 5 (71) 0 (0) 0 (0)	0 (0) 1 (14) 2 (29) 4 (57)	**0.004**

In parallel, the MRI results of the EVOO subjects were significantly different from the control group for both scores for short-term and long-term follow-up assessments (*p* = 0.017, *p* = 0.014, respectively) (Table 1).

**Fig. 2:**
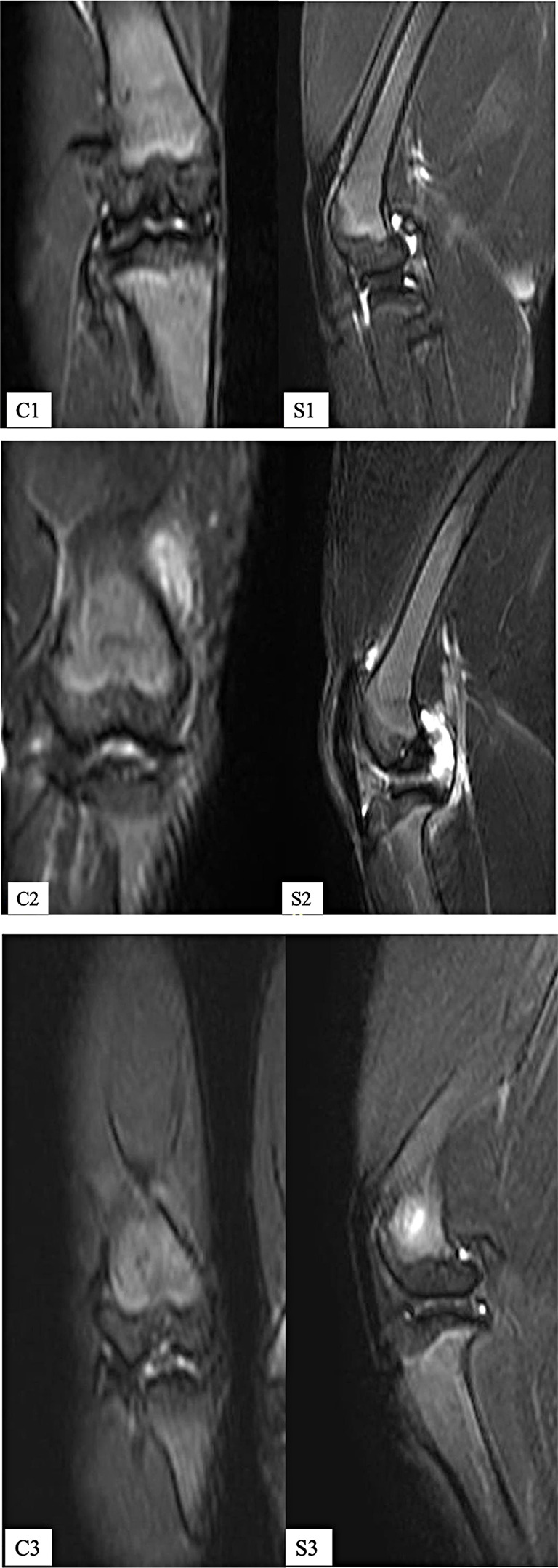
The coronal (**C**) and sagittal (**S**) images of a rabbit of the EVOO group. The images before the surgery (**C1** and **S1**). The images (**C2** and **S2**) were taken at the end of the fifth week with discontinuity in the cartilage. **C3** and **S3** show minimal signs of irregularity taken at the end of the study

The MRI images of the left knees of a rabbit of the EVOO group taken at the beginning, at the end of the fifth, and at the end of the 10th week of the study were shown in Fig. 2.

Mankin scoring results showed statistically significant differences between EVOO and the control group in the comparison of both total scores (*p* = 0.001 for the short term and *p* = 0.004 for the long term) (Table 1). In addition, there were statistically significant differences in histologic subgroup scoring, including macroscopic appearance, chondrocyte cell number, staining, and Tidemark integrity in both short-term (*p* = 0.005, *p* = 0.028, *p* = 0.001, *p* = 0.005, respectively) and long-term evaluations (*p* = 0.002, *p* = 0.014, *p* < 0.001, *p* = 0.200, respectively).

The microscopic observations revealed that hypercellular cartilage regions were observed in the hematoxylin-eosin-stained sections of the articular cartilage specimens of the EVOO group, especially near the damaged area. The chondrocytes forming a clone (isogeny) formation in the radial zone were notable. The proliferation of chondrocytes in the lacunae and the regular formation of extracellular matrix resulted in prominence in the tidemark zone, and a regular zone structure was observed. Superficial erosion decreased with new cartilage formation in the superficial zone.

**Fig. 3:**
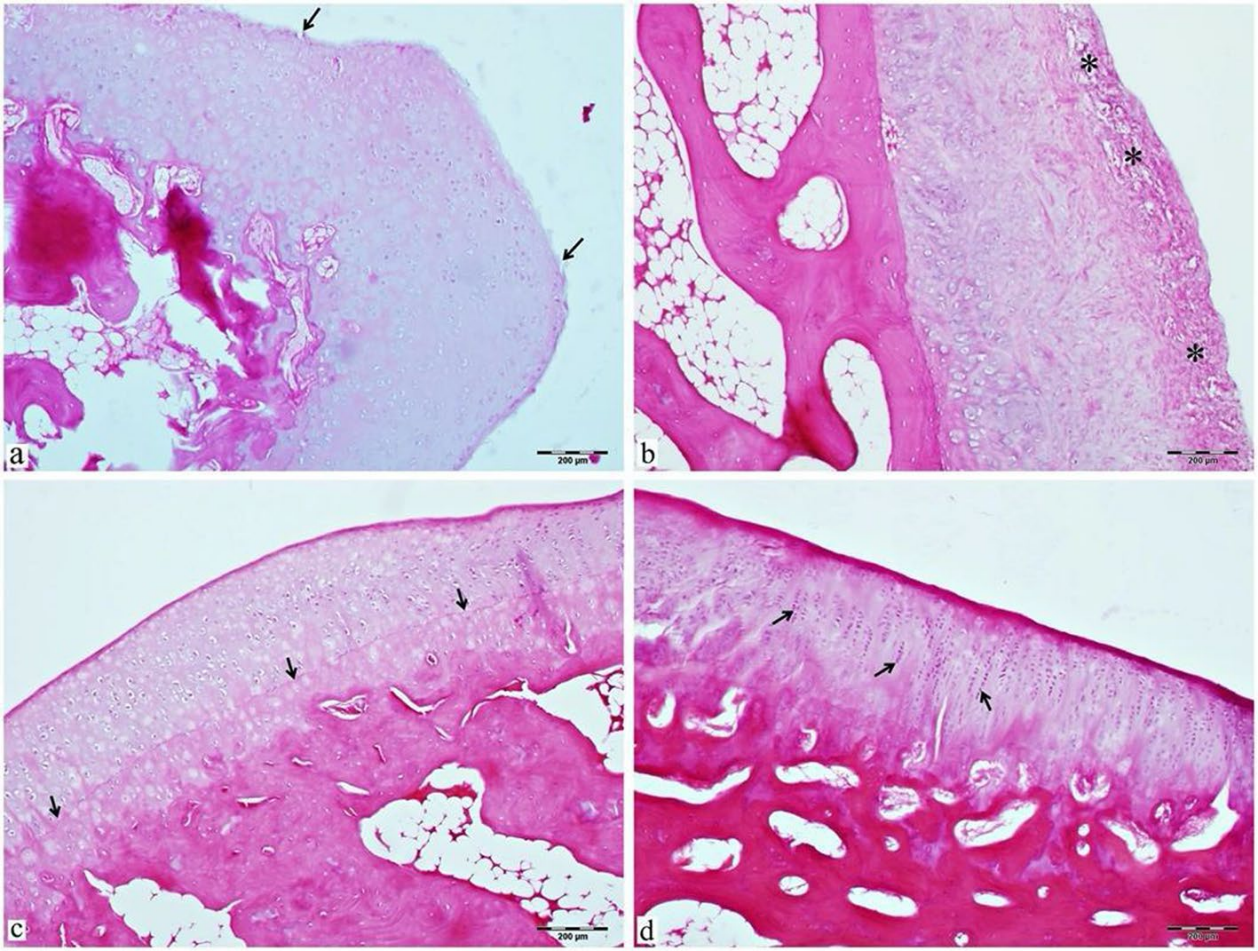
Light microscope images of (**a**, **b**) hematoxylin-eosin (H&E) stained articular cartilage sections of the control group with osteoarthritis and no treatment (**a**) Deterioration and limited superficial erosion (arrow) in the superficial layer of articular cartilage due to osteoarthritis, (**b**) focal fibrillation (*) in the superficial zone. Light microscope images of (**c**, **d**) hematoxylin-eosin (H&E) stained articular cartilage sections of the treatment group in which osteoarthritis was induced and EVOO treatment was administered. (**c**) After the treatment, the tidemark zone becomes prominent (arrow) (**d**); the resistance of the chondrocytes to compensation leads to the formation of clones (isogenic group) formation (arrow)

**Fig. 4:**
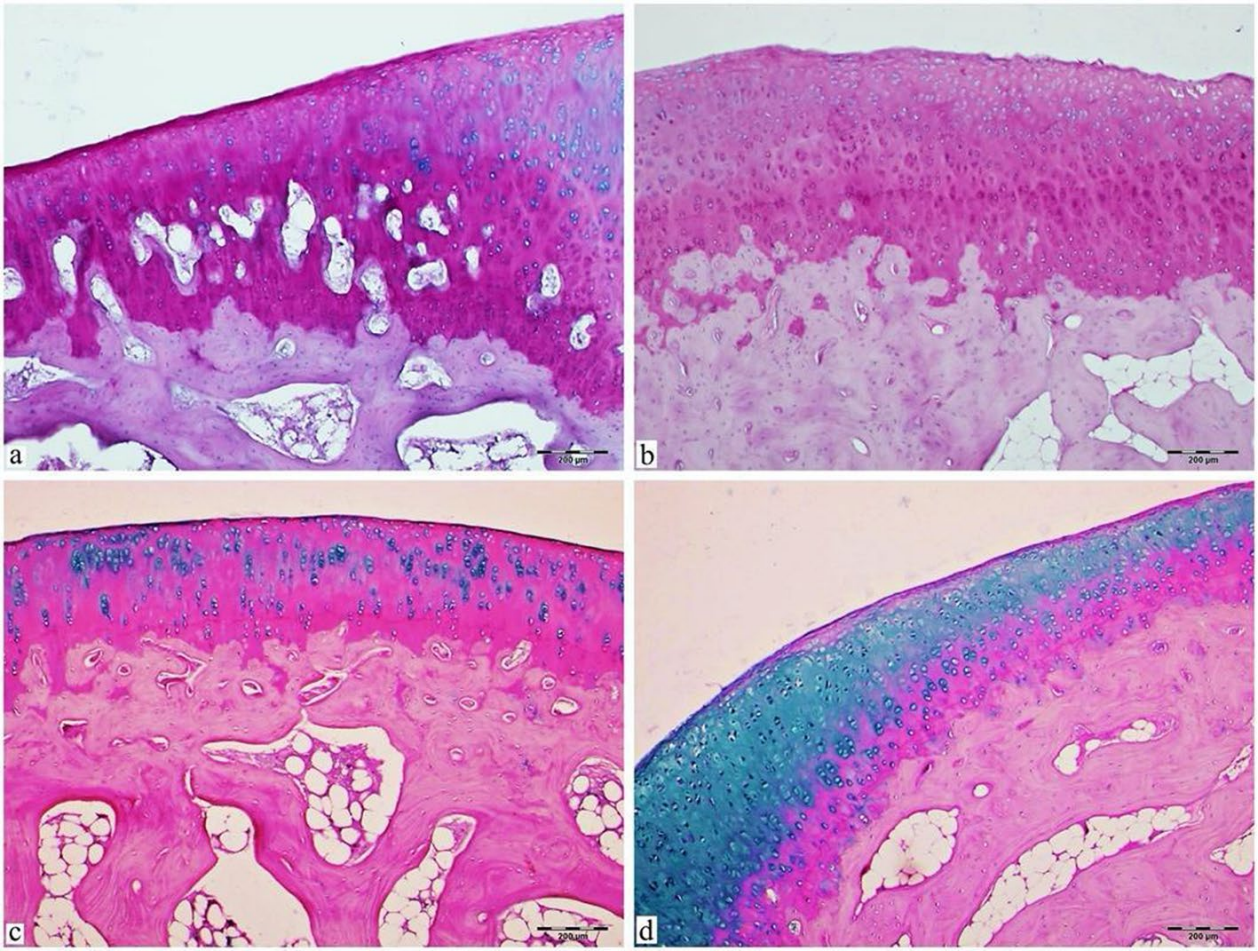
Light microscopy image of articular cartilage sections stained with Alcian blue/PAS. Alcian blue is specific for cartilaginous tissue, while dark pink periodic acid Schiff staining indicates subchondral bone. The articular cartilage of the experimental control group (**a**, **b**), in which osteoarthritis was induced and no treatment was administered, shows a limited area of new cartilage tissue stained with blue. Bar: 200𝜇m. In the articular cartilage of the treatment group (**c**, **d**), in which osteoarthritis was induced and EVOO treatment was applied, an increase in newly formed cartilage tissue stained in blue due to treatment is observed. Bar: 200𝜇m

In hematoxylin-eosin stained sections of articular cartilage specimens of the control group, disorganization of collagen arrangement in the cartilage matrix and focal fibrillation in the superficial zone were observed. There was no significant damage in the transitional and radial zones of the articular cartilage, whereas chondrocytes of the superficial zone were slightly deformed and irregular. It was noted that the chondrocyte organization and matrix arrangement in the tidemark zone of the articular cartilage were discontinuous, and the visibility of the tidemark zone, whose integrity was disrupted, was reduced. The subchondral bone appeared normal, with trabecular bone extensions and bone marrow cavities. The histological assessment findings of the cuts are presented in Figs. 3 and 4.

## Discussion

The literature currently lacks a definitive treatment for OA, and it appears that the search for better alternatives will continue, requiring innovative approaches. Our study has shown that compared to the control group, the rabbits administered intraarticular EVOO injection demonstrated promising results regarding gross morphological, radiological, and histological findings.

There are many reports that evidence the chondroprotective properties of EVOO with dietary administration, mainly attributed to the precious ingredient polyphenols. These compounds have antioxidant activity and are additionally shown to promote overall health due to anti-inflammatory, anti-allergic, anti-atherogenic, anti-thrombotic, and anti-mutagenic effects [17–19]. However, few studies focusing on intraarticular use have recently emerged, but they are still scarce.

An in vitro study comparing the outcomes of different polyphenols on articular cartilage degradation conducted by Natarajan et al. indicates that although the inflammation severity did not significantly differ, histomorphological scoring of the cartilage showed a substantial decrease in cartilage degradation, evidencing that intraarticular injections of polyphenols attach to cartilage and improve immunity to degradation. The study suggested that polyphenols administered via articular injection might have an extraordinarily valuable part in the protection of the cartilage in arthritis [20].

Regarding in vivo studies, Qin et al. assessed the chondroprotective effect of resveratrol, a polyphenol, on artificially induced OA in mice knees and found that intra-articular injection of resveratrol delayed articular cartilage degeneration [21]. Another study conducted by Reiter et al. on the administration of epigallocatechin-gallate in rat knees indicates that the injection of the polyphenol might develop strengthened tissue and delay or balance the matrix degradation caused by arthritis [9]. In another intraarticular resveratrol injection study, Elmalı et al. stated that intra-articular injections of the polyphenol into rabbit knees following artificially induced OA decreased the intensity of articular cartilage lesions and the inflammatory signs of the synovium [11]. In parallel to the studies, the morphological and histological evaluation of our EVOO-treated subjects showed that hypercellular cartilage regions were observed in the hematoxylin-eosin stained sections compared to the control group, especially near the damaged area. Radiological assessment of the EVOO group revealed that all subjects were scored in the first two grades with no to minimal breaks in the articular cartilage.

Furuuch et al., in 2018, suggested that polyphenol compounds protect cells from oxidative stress by significantly reducing ROS and NO levels [22]. Similarly, in our study, the evaluation of the EVOO group performed on the 10th week showed that the articular cartilage structure was histologically preserved, normal hemostasis of the cartilage was maintained, interpreted as indications of chondroprotective properties, with cartilage regeneration and restoration in the compressed areas, which were noteworthy.

### Limitations

The most important limitation of our study was that the agent EVOO itself was a combination of numerous essential chemicals, some of which play major roles in increasing the rate of cartilage tissue regeneration and accelerating healing. Such a broad approach may have shadowed the success of the primary substitutes of the compound. However, this could also be considered a strength of the study, as it also aimed to find a convenient agent that was accessible, available, and cheap for use in OA. Another major limitation was that the study was an animal experiment, and the effects on humans were of limited interpretability. Also, The amount of EVOO used on the subjects was decided based on the intraarticular volume of a rabbit knee which might prevent developing reasonable assumptions on the exact outcomes when used on human knees, since the volume and the surface area which the oil contacts differ greatly. Finally, the OA of the subjects was mechanically induced and can be most closely compared to spontaneous OA among human OA types.

## Conclusion

We have observed promising outcomes of the intra-articular application of EVOO in the treatment of acute degenerative osteoarthritis in rabbit knees. Due to its potential cartilage restorative and regenerative effects, EVOO, when administered intra-articularly, may be a promising agent to consider for further research in the treatment of OA.

## Data Availability

The data that support the findings of this study are available from the corresponding authors, AP and MYG, upon request.
